# A Narrative Review of Selective Versus Routine Histopathological Examination of Gallbladders in Low-Risk Patients

**DOI:** 10.7759/cureus.86609

**Published:** 2025-06-23

**Authors:** Sathyaseelan Arumugam, Shaurya Aggarwal

**Affiliations:** 1 General and Colorectal Surgery, Lister Hospital, East and North Hertfordshire Teaching NHS Trust, Stevenage, GBR

**Keywords:** cholangiocarcinoma, cholecystectomy, gallbladder cancer, histology, routine, selective, specimen’s

## Abstract

Gallbladder diseases are common surgical conditions that often necessitate a cholecystectomy. Traditionally, gallbladder specimens are sent for routine histopathological examination to detect incidental gallbladder carcinoma (IGBC). However, the need for this in low-risk patients remains a subject of debate, particularly considering the low incidence of IGBC and the strain on pathology services. This narrative review aimed to compare selective versus routine histopathological examination of gallbladder specimens in low-risk patients undergoing cholecystectomy. This review complies with the Preferred Reporting Items for Systematic reviews and Meta-Analyses (PRISMA) guidelines. A comprehensive search was conducted for studies between 2000 and 2022 across PubMed, Scopus, Embase, Web of Science, Cochrane Library, and ScienceDirect. Included studies involved patients with low preoperative suspicion of malignancy who were later diagnosed with gall bladder cancer via either routine or selective histopathological examination.

Sixteen studies met the inclusion criteria. Studies supporting selective histopathology reported that all cases of IGBC were suspected either preoperatively on imaging or intraoperatively based on macroscopic features. In contrast, studies advocating routine histology identified significant numbers of IGBC in patients with no intraoperative suspicion, including some with advanced-stage disease. These findings suggest that while selective examination may be safe in low-incidence regions with experienced surgical assessment, routine histology remains a more sensitive method in other areas. Selective histopathological examination of gall bladder specimens may be considered in carefully selected low-risk patients, provided a thorough intraoperative evaluation is performed. However, routine examinations remain important in regions with higher disease prevalence.

## Introduction and background

Cholecystectomy is one of the most commonly performed surgical procedures worldwide [1]. It is primarily indicated for symptomatic cholelithiasis [2]. Cholelithiasis affects approximately 10-15% of the population in developed countries, and despite being a benign condition, it is one of the main risk factors for gallbladder carcinoma (GBC) [3]. GBC is the fifth most prevalent cancer of the gastrointestinal system and the most common cancer of the biliary tract globally [4]. The incidence of GBC has significant geographic variation; it is approximately 1% in high-risk regions like India, Japan, Chile, and China, and approximately 0.4% in low-risk countries/regions such as Europe and the United States [5]. The updated joint guidelines developed between the European Society of Gastrointestinal and Abdominal Radiology (ESGAR), European Association for Endoscopic Surgery and other Interventional Techniques (EAES), International Society of Digestive Surgery-European Federation (EFISDS) and European Society of Gastrointestinal Endoscopy (ESGE), which were published in 2022 categorises the risk factors for gall bladder cancers as follows: age more than 60 years, primary sclerosing cholangitis, Asian ethnicity and sessile polypoidal lesions including focal gall bladder thickening >4 mm [6].

Gallbladder cancer generally has a poor prognosis, with survival largely reliant on early identification. While the five-year survival rate is only 5% overall, it increases to 79% when detected early [7]. Incidental gallbladder carcinoma (IGBC) refers to gallbladder cancer diagnosed histologically after cholecystectomy is performed for presumed benign conditions, without prior clinical or intraoperative suspicion. It is also known as occult, inapparent, or missed GBC [5]. The reported incidence of IGBC ranges from 0.2% to 2.9% [7]. When gallbladder cancer is unexpectedly detected, overall survival depends on the T staging. For Tis and T1a, simple cholecystectomy is sufficient. Further advanced stages will require extended surgery [7]. The choice between routine and selective histopathological evaluation of gallbladder specimens to detect IGBC remains a debated issue in countries with differing GBC incidence rates. Routine histological evaluation is frequently advised because of the tumour's aggressive nature and the benefits of early identification. However, the increasing volume of cholecystectomies and the relatively low incidence of IGBC have prompted discussions around a selective approach. The selective criteria, however, must ensure that patient safety is not compromised [3].

With the increasing number of cholecystectomies performed annually across the NHS, there has been a proportional rise in gallbladder specimens referred for histopathological examination. This growing volume places considerable pressure on pathology services and contributes to rising healthcare expenditure. Hence, it is important to evaluate the clinical necessity and safety of routine histopathological analysis compared to a selective approach. This evaluation is vital to ensure the effective allocation of healthcare resources without compromising diagnostic accuracy or patient outcomes. This narrative review aims to explore the outcomes of selective versus routine histological evaluation of gallbladder specimens in low-risk patients undergoing cholecystectomy for benign gallbladder disease. We aim to explore if selective histopathology can replace routine histopathology in these patients while maintaining patient safety.

## Review

Materials and methods

Study Design and Ethical Considerations

We adopted a narrative review design. Ethics committee approval was not required, given that this study represents a synthesis of published data, and it did not involve direct patient or public participation.

Sources of Information and Search Methodology

A comprehensive search was conducted from 2000 to 2022 across several databases, including PubMed, Google Scholar, Scopus, ScienceDirect, and Web of Science. The search utilised the keywords [(‘gallbladder cancer’ or ‘cholecystectomy’ or ‘incidental carcinoma of the gallbladder’ or ‘routine histology’ or ‘selective histology’)]. The snowball technique was used by reviewing the reference lists of included studies to identify any relevant and eligible studies that were not identified in the initial search.

Inclusion and Exclusion Criteria

English-language publications from 2000-2022 were considered in this review. Case reports, literature reviews, and animal trials were excluded. Chinese and Indian populations, in whom the incidence of gall bladder cancer is high compared to the rest of the world, as well as studies with insufficient or superfluous data, were also excluded. We aimed to include studies involving individuals who underwent the procedure for benign gallbladder diseases and whose histological examination later revealed incidental gallbladder cancer.

Study Selection

**Figure 1:**
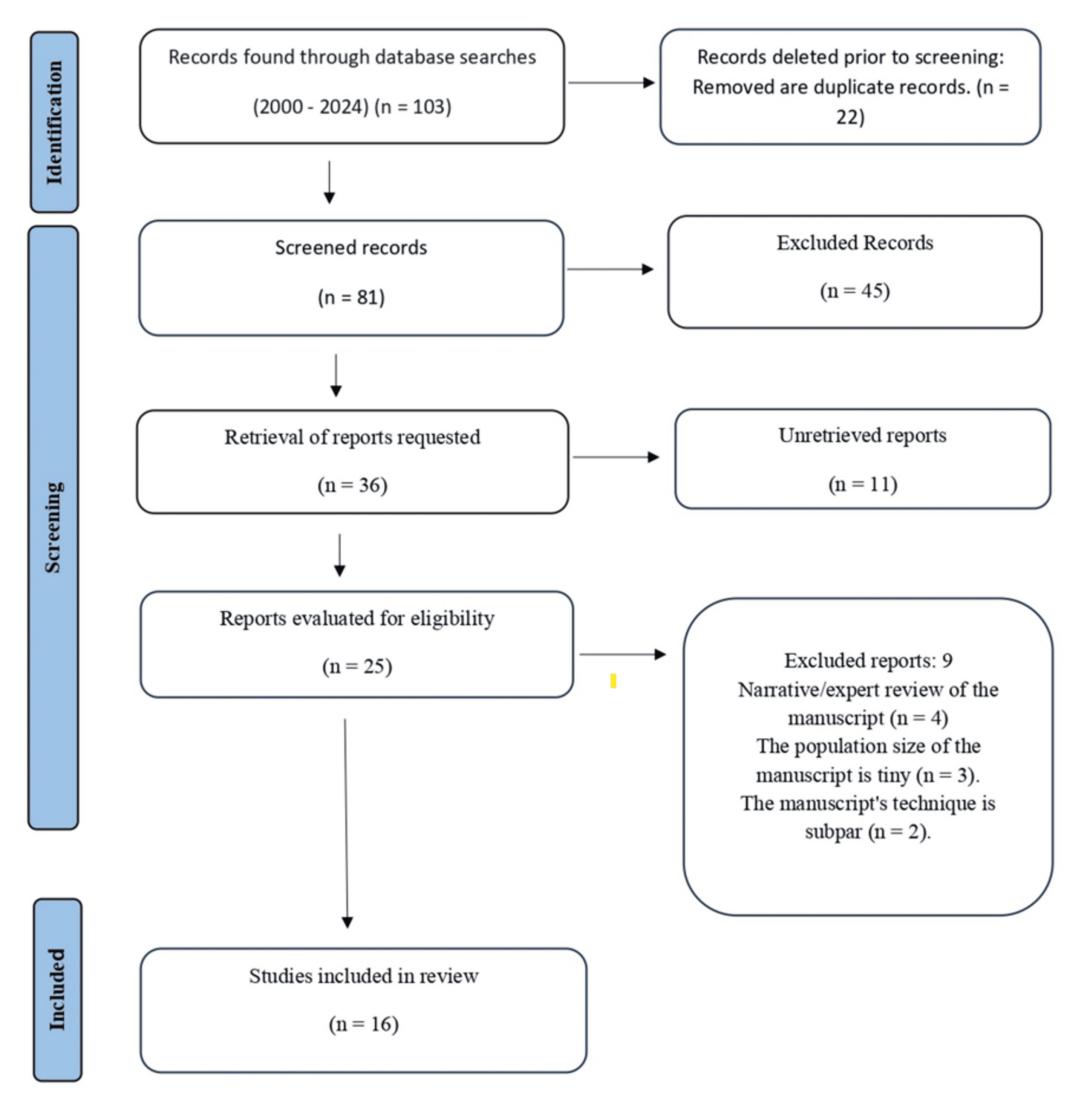
PRISMA flow chart depicting the selection of studies PRISMA: Preferred Reporting Items for Systematic reviews and Meta-Analyses

The titles and abstracts of all the studies were screened. Studies that did not meet the inclusion criteria were removed. The search results, as well as the process of screening and selection of studies, are presented in the Preferred Reporting Items for Systematic reviews and Meta-Analyses (PRISMA) flow diagram (Figure 1). Any disputes were resolved by discussion within the research team. The included studies were reviewed by two independent reviewers.

Data Collection Process

As part of the data collection procedure, key information was extracted from each article, cross-checked, and validated. The data gathered included study guidelines, the country, the year, the total number of cholecystectomies, the total number of GBC cases, the percentage of gallbladders with concerned intraoperative findings for GBC, the percentage of IGBC cases that were unexpectedly detected by histology, and the identity of the author. Additionally, information about the gross morphological features or intraoperative features was obtained.

Evaluation of Study Quality

The Newcastle-Ottawa Scale, which uses values ranging from 0 to 9 to evaluate selection, comparability, and outcome assessment, was used by the authors to evaluate the research's quality. The main outcome measure was as follows: histologically detecting IGBC following cholecystectomy for benign gallbladder disorders.

Results

Study Selection

The study selection process outlines the step-by-step method used to identify and select studies for inclusion. Initially, 103 records were identified through database searches. After the removal of 22 duplicates, 81 records were screened based on titles and abstracts. Of these, 45 were excluded for not meeting the inclusion criteria. Of the remaining 36 selected for retrieval, 11 records could not be obtained. As a result, 25 records were evaluated for eligibility, with nine being excluded for specific reasons: four were narrative or expert reviews, three had a small population size, and two demonstrated poor methodology. Ultimately, 16 studies were included in the review. A flowchart visually representing the filtering process from initial identification to final inclusion is presented in Figure 1.

This narrative review analysed 16 studies on both selective and routine histopathological examination in gall bladder carcinoma. The review's studies are all in English and were carried out in several nations with varying rates of GBC. With ratings ranging from 0 to 9, the Newcastle-Ottawa Scale, which assesses selection, comparability, and outcome evaluation, was used to evaluate the research's quality. Research with a high risk of bias was excluded from the review; only high-quality research was included. The studies collectively examined approximately 60,000 cholecystectomy specimens. The majority were retrospective in design, with only two prospective studies. Most studies were from countries with low to moderate incidence of gallbladder cancer, including the United Kingdom, Sweden, Libya, Malaysia, Mexico, Sri Lanka, Nepal, and Pakistan.

Selective Histopathology

Ten of the included studies supported selective histopathological examination of gallbladder specimens. These studies did not find any IGBCs on histology. All cases of confirmed gall bladder malignancy had either been suspected preoperatively or demonstrated suspicious features intraoperatively [8-17]. For example, Benkhadoura et al. in Libya reported four cases of gallbladder malignancy among 3,423 cholecystectomy specimens. Two were anticipated intraoperatively, and two were suspected on imaging; none were unexpected histological discoveries. Similarly, Talreja et al. in Pakistan found that all 11 IGBC cases out of a total of 964 patients had intraoperative features suspicious of malignancy.

Chin et al. from Malaysia reported no unexpected histological findings in their seven cases of GBM. Likewise, Dezoysa et al. from Sri Lanka also found no histological surprises in their four cases of GBM. In a study conducted in the United Kingdom, Emmett et al. found no unexpected histological findings in 12 patients with GBM, all of whom were suspected intraoperatively. Romero et al. evaluated cases in a prospective trial in Mexico that included both pathologists and surgeons. All three GBM cases (3/3) were initially suspected intraoperatively by surgeons and later confirmed by pathologists during gross examination.

Routine Histopathology

Six studies favoured routine histological analysis due to high rates of IGBC being detected on histology alone [18-21,2,7]. According to Lundgren et al. in Sweden (the study with the biggest sample size of all the included studies, involving 36,010 cholecystectomy specimens), 153 of 213 cases were detected solely via routine histological analyses. Similarly, nine out of 20 instances of GB were only discovered through histological investigation, according to Shrestha et al. from Nepal [18]. According to Patel et al. from the United Kingdom, histological evaluation of all six GB cases (6/6) revealed unexpected results, including two T3 lesions, two T2 lesions, and one T1b lesion. In the study by Siddiqui et al. from Pakistan, six out of 12 patients with IGBC were only discovered on histopathology and had no macroscopic abnormalities on intraoperative assessment. Ghimire et al. similarly found 10 out of 10 cases of IGBC among 783 specimens with no intraoperative suspicion. Table 1 summarises the characteristics of the included studies.

**Table 1:** Characteristics of included studies GBC: gallbladder carcinoma

Study	Country	Design	No. of gall bladders	No. of GBC	GBC suspected preoperatively	GBC suspected intraoperatively	GBC diagnosed incidentally	Recommendation
Dix et al. (2003) [12]	UK	Retrospective	1,292	5	3	2	0/5	Selective
Samad (2005) [21]	Pakistan	Retrospective	1,396	16	3	8	5/16	Routine
Darmas et al. (2007) [11]	UK	Retrospective	1,452	4	1	3	0/4	Selective
De Zoysa et al. (2010) [10]	Sri Lanka	Retrospective	477	4	2	2	0/4	Selective
Shrestha et al. (2010) [18]	Nepal	Retrospective	570	20	6	5	9/20	Routine
Ghimire et al. (2011) [20]	Nepal	Retrospective	783	10	0	0	10/10	Routine
Byars and Pursnani (2012) [8]	UK	Retrospective	2,696	7	5	2	0/7	Selective
Chin et al. (2012) [17]	Malaysia	Retrospective	1,375	7	5	2	0/7	Selective
Romero-González et al. (2012) [16]	Mexico	Prospective	150	3	2	1	0/3	Selective
Sajjad et al. (2012) [14]	Pakistan	Retrospective	326	2	2	2	0/2	Selective
Siddiqui et al. (2013) [19]	Pakistan	Prospective	220	6	0	6	6/12	Routine
Emmett et al. (2015) [13]	UK	Retrospective	4,776	12	0	12	0/12	Selective
Patel et al. (2016) [2]	UK	Retrospective	4,027	6	0	0	6/6	Routine
Talreja et al. (2016) [15]	Pakistan	Retrospective	964	11	0	11	0/11	Selective
Lundgren et al. (2018) [7]	Sweden	Retrospective	36,010	213	0	60	153/213	Routine
Benkhadoura et al. (2019) [9]	Libya	Retrospective	3423	4	2	2	0/4	Selective

Methodological Quality and Bias Profile of Included Studies

The assessment of bias in the 16 included studies, based on the ROBINS-I (Risk of Bias in Non-randomised Studies of Interventions) tool, highlights a shared methodological limitation. Specifically, Domain 1: Confounding was rated as "moderate risk" across all studies. This points to how confounding factors were handled. Confounding bias arises when variables, such as age, comorbidities, or disease severity, influence both the likelihood of receiving an intervention and the outcomes of interest, leading to biased estimates if not properly controlled.

Despite the issue of confounding, the studies generally performed well in the other domains assessed by ROBINS-I. In Domains 2 through 7, most studies were rated as "low risk," indicating that these studies were robust in terms of participant selection, classification of interventions, adherence to the intended interventions, handling of missing data, outcome measurement, and selective reporting. These findings suggest that the studies employed sound methodology in all areas except for confounding. However, there was an exception. Romero-González et al. [16] received a "moderate risk" rating in Domain 6: Measurement of Outcomes, indicating potential weaknesses in the way outcomes were measured. This may include factors like a lack of blinding in outcome assessment or the use of subjective measures. Table 3 summarises the assessment of risk of bias in the included studies.

**Table 2:** Overview of the risk of bias in included studies

Risk of bias assessment								
S. no	Study	Domain 1	Domain 2	Domain 3	Domain 4	Domain 5	Domain 6	Domain 7
1	Dix et al. [12]	Moderate risk	Low risk	Low risk	Low risk	Low risk	Low risk	Low risk
2	Samad [21]	Moderate risk	Low risk	Low risk	Low risk	Low risk	Low risk	Low risk
3	Darmas et al. [11]	Moderate risk	Low risk	Low risk	Low risk	Low risk	Low risk	Low risk
4	De Zoysa et al. [10]	Moderate risk	Low risk	Low risk	Low risk	Low risk	Low risk	Low risk
5	Shrestha et al. [18]	Moderate risk	Low risk	Low risk	Low risk	Low risk	Low risk	Low risk
6	Ghimire et al. [20]	Moderate risk	Low risk	Low risk	Low risk	Low risk	Low risk	Low risk
7	Byars and Pursnani [8]	Moderate risk	Low risk	Low risk	Low risk	Low risk	Low risk	Low risk
8	Chin et al. [17]	Moderate risk	Low risk	Low risk	Low risk	Low risk	Low risk	Low risk
9	Romero-González et al. [16]	Moderate risk	Low risk	Low risk	Low risk	Low risk	Moderate risk	Low risk
10	Sajjad et al. [14]	Moderate risk	Low risk	Low risk	Low risk	Low risk	Low risk	Low risk
11	Siddiqui et al. [19]	Moderate risk	Low risk	Low risk	Low risk	Low risk	Low risk	Low risk
12	Emmett et al. [13]	Moderate risk	Low risk	Low risk	Low risk	Low risk	Low risk	Low risk
13	Patel et al. [2]	Moderate risk	Low risk	Low risk	Low risk	Low risk	Low risk	Low risk
14	Talreja et al. [15]	Moderate risk	Low risk	Low risk	Low risk	Low risk	Low risk	Low risk
15	Lundgren et al. [7]	Moderate risk	Low risk	Low risk	Low risk	Low risk	Low risk	Low risk
16	Benkhadoura et al. [9]	Moderate risk	Low risk	Low risk	Low risk	Low risk	Low risk	Low risk

Discussion

Gall bladder cancer has a high mortality rate due to its ineffective treatment, strong propensity to spread, and delayed presentation. Growing awareness and easier access to medical facilities may be the cause of the rising early detection rates. However, it remains challenging to identify gall bladder cancer in its early stages using radiological and clinical methods. Only when a substantial intraluminal mass is present may 40-60% of cases be identified during ultrasonography (USG) evaluation. Since diffuse wall thickening is also observed in chronic inflammatory conditions, it is not a specific finding for malignancy [22]. Patient factors, clinical features, and the surgeon's intraoperative evaluation of the gallbladder can guide the likelihood of gallbladder carcinoma, but are not definitive.

This review considered whether combined criteria based on patient factors, imaging, and intraoperative findings can be used to safely omit some gall bladder specimens from histopathological analysis. Such criteria must ensure no cases of GBC are missed [23]. Studies supporting selective histology in this review reported that all diagnosed malignancies were anticipated on imaging or intraoperative findings. They conclude that the probability of cancer in a gallbladder with a normal macroscopic appearance is low, and that histopathological examination may be unnecessary [8,10,13]. This is particularly true in low-risk populations. Another consideration is the growing demand for pathology services. In resource-limited settings, where constraints are both financial and include a shortage of histopathologists, as highlighted by De Zoysa et al. [10], a selective policy could help alleviate pressure on the system.

Advocates of routine histopathological assessment emphasise its role in detecting subclinical malignancies and facilitating follow-up treatment in cases of IGBC. According to Agarwal et al., early diagnosis through routine histology was associated with better prognosis. In contrast, patients who did not undergo histopathological evaluation were often diagnosed at a more advanced stage. These findings highlight the significance of meticulous intraoperative evaluation and consideration of regional GBC incidence when determining histological protocols [24]. Several studies have shown that even advanced-stage cancers, T2 and above, can occur without any visible macroscopic abnormalities [2,14,20]. In such cases, relying solely on intraoperative assessment risks missing cancers that require further treatment beyond cholecystectomy. The omission of histopathology in these patients may miss the window of curative treatment and lead to avoidable morbidity and mortality. Furthermore, the medicolegal implications of these cases may outweigh the cost savings from selective examination.

Malignant gallbladder appears normal or shows only subtle suspicious findings intraoperatively; the disease is usually at an early stage, such as Tis or T1a, which does not necessitate any treatment beyond simple cholecystectomy. When choosing the histological evaluation of gallbladder specimens, a thorough intraoperative examination is crucial for detecting incidental gallbladder cancer and determining the local incidence of gallbladder cancer [3].

## Conclusions

The incidence of incidental gallbladder cancer discovered by histology is considerably on decline, thanks to careful intraoperative macroscopy. In light of this, cholecystectomy tissues should be histologically examined selectively, especially in areas where gallbladder cancer is uncommon. Several published studies suggest that most gallbladder cancers could be detected by simply opening the specimen in the operating room during surgery. Therefore, if a selective approach is adopted, the decision to send the gallbladder for histopathological examination should be based on a macroscopic evaluation of the opened specimen and the presence of any risk factors discussed above.
